# In Vitro Inhibition of Renal OCT2 and MATE1 Secretion by Antiemetic Drugs

**DOI:** 10.3390/ijms22126439

**Published:** 2021-06-16

**Authors:** Blessy George, Xia Wen, Edgar A. Jaimes, Melanie S. Joy, Lauren M. Aleksunes

**Affiliations:** 1 Department of Pharmacology and Toxicology, Ernest Mario School of Pharmacy, Rutgers University, 170 Frelinghuysen Road, Piscataway, NJ 08854, USA; bless.george@gmail.com (B.G.); wen@eohsi.rutgers.edu (X.W.); 2 Renal Service, Memorial Sloan Kettering Cancer Center, New York, NY 10065, USA; jaimese@mskcc.org; 3 Department of Pharmaceutical Sciences, Skaggs School of Pharmacy and Pharmaceutical Sciences, University of Colorado, Aurora, CO 80045, USA; melanie.joy@cuanschutz.edu; 4 Cancer Center, University of Colorado, Aurora, CO 80045, USA; 5 Division of Renal Diseases and Hypertension, School of Medicine, University of Colorado, Aurora, CO 80045, USA; 6 Environmental and Occupational Health Sciences Institute, Piscataway, NJ 08854, USA

**Keywords:** OCT2, MATE1, cationic, 5-HT_3_ antagonist, kidney, secretion, transport

## Abstract

The organic cation transporter 2 (OCT2) and multidrug and toxin extrusion protein 1 (MATE1) mediate the renal secretion of drugs. Recent studies suggest that ondansetron, a 5-HT_3_ antagonist drug used to prevent nausea and vomiting, can inhibit OCT2- and MATE1-mediated transport. The purpose of this study was to test the ability of five 5-HT_3_ antagonist drugs to inhibit the OCT2 and MATE1 transporters. The transport of the OCT2/MATE1 probe substrate ASP^+^ was assessed using two models: (1) HEK293 kidney cells overexpressing human OCT2 or MATE1, and (2) MDCK cells transfected with human OCT2 and MATE1. In HEK293 cells, the inhibition of ASP^+^ uptake by OCT2 listed in order of potency was palonosetron (IC_50_: 2.6 μM) > ondansetron > granisetron > tropisetron > dolasetron (IC_50_: 85.4 μM) and the inhibition of ASP^+^ uptake by MATE1 in order of potency was ondansetron (IC_50_: 0.1 μM) > palonosetron = tropisetron > granisetron > dolasetron (IC_50_: 27.4 μM). Ondansetron (0.5–20 μM) inhibited the basolateral-to-apical transcellular transport of ASP^+^ up to 64%. Higher concentrations (10 and 20 μM) of palonosetron, tropisetron, and dolasetron similarly reduced the transcellular transport of ASP^+^. In double-transfected OCT2-MATE1 MDCK cells, ondansetron at concentrations of 0.5 and 2.5 μM caused significant intracellular accumulation of ASP^+^. Taken together, these data suggest that 5-HT_3_ antagonist drugs may inhibit the renal secretion of cationic drugs by interfering with OCT2 and/or MATE1 function.

## 1. Introduction

Renal secretion is achieved by the coordinated uptake and efflux of drugs across the tubule epithelium. For drugs and toxicants that are organic cations, their transepithelial transport into the filtrate is first achieved by the organic cation transporter 2 (OCT2) on the basolateral surface and, subsequently, by the multidrug and toxin extrusion 1 (MATE1) transporter on the brush border membrane. Prior to discovering the OCT2/SLC22A1 and MATE1/SLC47A1 genes, it was well understood that the secretion of organic cations could be inhibited pharmacologically [1]. Using a variety of experimental approaches across different preclinical species, it was demonstrated that organic cations undergo active transport and renal secretion [2,3,4]. Since these early observations, the research has advanced to understand not only the molecular mechanisms of organic cation secretion but also the potential for clinically-relevant drug interactions following the disruption of OCT2 and MATE1 (reviewed in [5]).

Antagonists of the serotonin 5-HT_3_ receptor have emerged as critical drugs for the management of nausea and vomiting. Serotonin is released by the enterochromaffin cells of the small intestine. 5-HT_3_ antagonists inhibit the ionotropic ligand-gated ion channel on afferent nerves from the vagus and prevent the vomiting reflex [6]. Ondansetron was the first 5-HT_3_ antagonist approved to prevent chemotherapy-induced nausea and vomiting [7,8]. Since that time, additional drugs in this class (including tropisetron, granisetron, dolasetron, and palonosetron) have been developed [9]. While the primary therapeutic indication of 5-HT_3_ antagonists has been the prevention of chemotherapy and radiation induced emesis, they are also approved for the management of postoperative nausea and vomiting and are used off-label to treat morning sickness, hyperemesis gravidarum, postoperative delirium, and pruritus [10,11,12,13,14].

**Figure 1:**
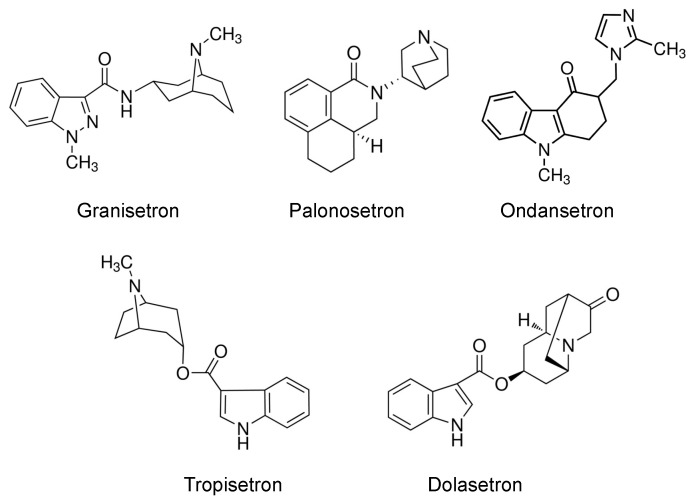
Structure of 5-HT_3_ antagonists.

Given the cationic nature of 5-HT_3_ antagonists (Figure 1), they have emerged as substrates and inhibitors of OCT and MATE transporters. In vitro studies have revealed that ondansetron and tropisetron are substrates and inhibitors of OCT1 and OCT2 [15,16,17,18,19,20]. Moreover, individuals with loss-of-function variants in the OCT1/SLC22A1 gene have been shown to have altered tropisetron pharmacokinetics and improved clinical efficacy [16]. Likewise, ondansetron can inhibit MATE transporters, leading to renal accumulation of the antidiabetic drug metformin, as well as increased nephrotoxicity of the anticancer drug cisplatin [19]. In humans, ondansetron decreases the renal clearance of metformin, presumably by inhibiting MATE1 efflux [21]. Together, these data point to the potential for 5-HT_3_ antagonists to inhibit the transepithelial secretion of drugs via OCT2 and MATE1. Therefore, we sought to systematically compare five 5-HT_3_ antagonists for their ability to inhibit human OCT2 and MATE1-mediated transport of a probe cationic substrate using single- and double-transfected kidney cells. Of note, the current study focused primarily on MATE1, as levels of MATE2-K protein in the human kidney cortex have been previously shown to be below the lower limit of quantification using mass spectrometry [22].

## 2. Results

### 2.1. Characterization of ASP^+^ as a Fluorescent Substrate in HEK293 Cells Overexpressing OCT2 and MATE1

**Figure 2:**
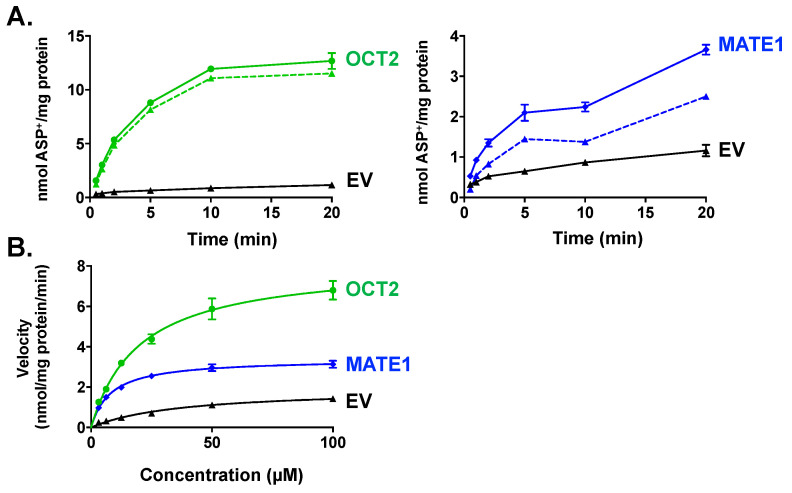
Time and Concentration-Dependent Accumulation of ASP^+^ in HEK293 Cells Overexpressing OCT2 or MATE1. HEK cells expressing empty vector (EV), OCT2, or MATE1 were incubated for different time periods (0.5–20 min, 10 μM ASP^+^, 37 °C) to evaluate time-dependent uptake (**A**) or with increasing concentrations of ASP^+^ (0–100 μM, 1 min 37 °C) for concentration-dependent uptake (**B**) on a shaker. Specific transport uptake is shown in dashed lines. Intracellular fluorescence was quantified and normalized to protein concentration. Data are presented as mean ± SE (*n* = 3).

Initial studies characterized the uptake of ASP^+^ into cells overexpressing an empty vector (EV), OCT2, or MATE1 (Figure 2). ASP^+^ displayed time-dependent (Figure 2A) and concentration-dependent (Figure 2B) uptake in both OCT2- and MATE1-expressing cells and exhibited saturable kinetics (OCT2: V_max_ 8.1 nmol/mg/min, K_m_ 2.9 μM, R^2^ 0.95; MATE1: V_max_ 3.4 nmol/mg/min, K_m_ 8.2 μM, R^2^ 0.96). EV cells exhibited minimal accumulation of ASP^+^ (V_max_ 1.9 nmol/mg/min, K_m_ 37.3 μM, R^2^ 0.92). Based on these findings, ASP^+^ uptake was quantified after 1 min (linear range for OCT2 and MATE1 transport) at a concentration of 10 μM, which provided sufficient sensitivity for fluorescence detection and screening of 5-HT_3_ antagonists as inhibitors.

**Figure 3:**
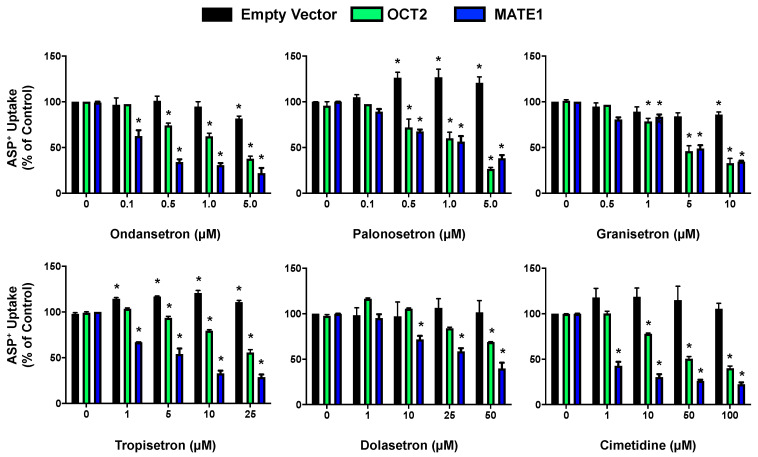
5-HT_3_ Antagonist Inhibition of ASP^+^ Transport in HEK293 Cells Overexpressing OCT2 and MATE1 following 5-HT_3_ Antagonist Treatment. Cells were incubated with ASP^+^ (10 μM) in the presence and absence of various concentrations of 5-HT_3_ antagonist or the positive control inhibitor, cimetidine. Fluorescence was quantified and normalized to protein concentration. Fluorescence quantified in empty vector, OCT2, and MATE1 treated with vehicle control (no inhibitor) was set to 100%. Data are expressed as mean ± SE (*n* = 3). * *p* < 0.05 compared to the vehicle.

To ensure these conditions reflected active transport by each transporter, the IC_50_ values of cimetidine, a well-established OCT2 and MATE1 inhibitor, were determined (Figure 3 and Table 1). The IC_50_ for cimetidine was 24.5 ± 4.0 μM in OCT2-expressing cells and 0.23 ± 0.2 μM in MATE1-expressing cells, in agreement with published data showing inhibition of MATE1 at lower concentrations [18,20]. Cimetidine had no influence on ASP^+^ uptake in EV cells.

### 2.2. Inhibition of OCT2- and MATE1-Mediated Transport by Antiemetic Drug in HEK293 Cells

Five different 5-HT_3_ antagonists (ondansetron, palonosetron, granisetron, tropisetron, and dolasetron) were evaluated for their inhibition of OCT2 and MATE1 transport in HEK293 cells using ASP^+^ as a substrate (Figure 3). A concentration-dependent decrease in ASP^+^ uptake was observed in OCT2- and MATE1-expressing cells in the presence of all five 5-HT_3_ antagonists tested across a range of concentrations. IC_50_ values for the inhibition of ASP^+^ accumulation by 5-HT_3_ antagonists using the concentration ranges tested are shown in Table 1. With the exception of granisetron, the other 5-HT_3_ antagonists inhibited MATE1 more potently than they did OCT2. OCT2-mediated transport was inhibited up to ~90% while MATE1-mediated transport was inhibited up to ~70% at the concentrations tested.

In general, the uptake of ASP^+^ by EV cells was not altered to a large degree by the 5-HT_3_ antagonists. However, it was noted that palonosetron and tropisetron stimulated additional ASP^+^ uptake in EV cells and the highest concentration of granisetron caused a small decrease in ASP^+^ accumulation.

### 2.3. Characterization of the Transcellular Transport and Intracellular Accumulation of ASP^+^ in OCT2/MATE1-Expressing MDCK Cells

**Figure 4:**
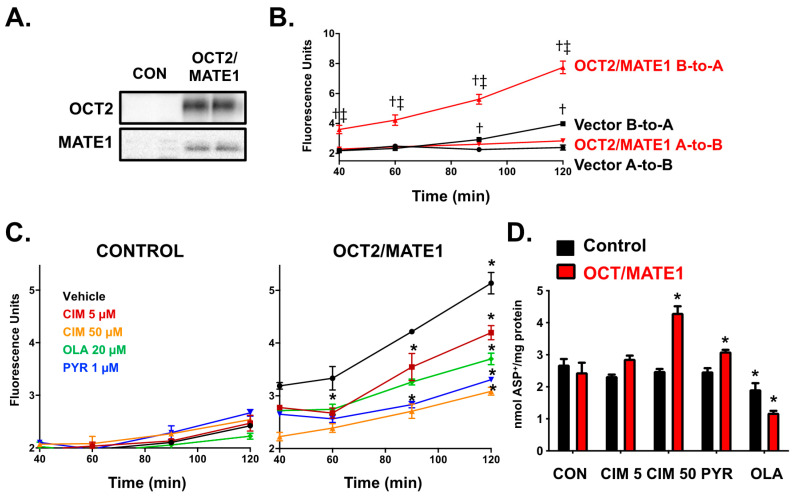
Transcellular Flux of ASP^+^ in Control and OCT2/MATE1-Transfected MDCK cells. (**A**). Protein expression of OCT2 (~63 kDa,) and MATE1 (~54 kDa) in vector control MDCK cells (lanes 1–2) and OCT2/MATE1 double transfected (lanes 3–4). (**B**). Cells were incubated with ASP^+^ (25 μM) in either apical or basolateral media for 120 min and fluorescence in apical or basolateral media was quantified (A-to-B: apical-to-basolateral; B-to-A: basolateral-to-apical). ^†^ *p* < 0.05 compared to A-to-B. ^‡^ *p* < 0.05 compared to vector. (**C**). Cells were incubated with ASP^+^ (25 μM) in basolateral media and positive control inhibitors in apical and basolateral media for 120 min. Fluorescence was measured in the apical chamber. (**D**). Intracellular fluorescence was quantified and normalized to protein concentration. Data are expressed as mean ± SE (*n* = 3). * *p* < 0.05 compared to no inhibitor. CIM–Cimetidine; PYR–Pyrimethamine; OLA–Olanzapine.

To investigate the combined contribution of OCT2 and MATE1 in transepithelial secretion, subsequent experiments were performed in MDCK cells that polarize with basolateral (OCT2) and apical (MATE1) surfaces. The expression of the OCT2 and MATE1 protein was confirmed in double-transfected MDCK cells using Western blotting (Figure 4A). The transcellular transport of the cationic probe substrate ASP^+^ (25 μM) was tested in these cells using Transwell inserts. The basolateral-to-apical (B-to-A) transport of ASP^+^ was much greater (up to 2.8-fold at 120 min) than the apical-to-basolateral (A-to-B) transport in the OCT2/MATE1 double transfected cells (Figure 4B). The B-to-A/A-to-B efflux ratio at 120 min was estimated to be 2.7 for OCT2/MATE1 cells supporting the active secretion of ASP^+^. In contrast, control cells exhibited much lower ASP^+^ transport in both directions compared to OCT2/MATE1 cells. The B-to-A transport of ASP^+^ was only significantly higher compared to the A-to-B transport in control cells at 90 (1.3-fold) and 120 min (1.7-fold). All further inhibition assays were performed in the B-to-A direction.

The ability of chemicals to inhibit ASP^+^ flux and accumulate in OCT2/MATE1 double transfected cells at the specified test conditions was assessed using three positive control inhibitors (cimetidine 5 and 50 μM, pyrimethamine 1 μM, and olanzapine 20 μM) (Figure 4C). Cimetidine is a known inhibitor of OCT2 and MATE1, with a greater potency for MATE1 (Table 1) [18]. Pyrimethamine is a specific MATE1 inhibitor [23], whereas olanzapine was found to inhibit OCT2 more potently (Supplementary Figure S1) [18,20]. All three chemicals inhibited the transcellular flux of ASP^+^ (18% cimetidine 5 μM, 40% cimetidine 50 μM, 36% pyrimethamine 1 μM, and 28% olanzapine 20 μM at 120 min).

The accumulation of ASP^+^ intracellularly was also quantified in lysates at 120 min. Cimetidine and pyrimethamine increased the intracellular accumulation of ASP^+^ by 1.8-fold and 1.3-fold (due to inhibition of MATE1 primarily), respectively, whereas olanzapine decreased the intracellular accumulation of ASP^+^ by 51% (due to the inhibition of OCT2). In control cells, no significant inhibition of B-to-A transport of ASP^+^ was observed at any of the time points. In control cells, there were also no significant changes in intracellular accumulation of ASP^+^ compared to vehicle control cells, except for a modest decrease in ASP^+^ accumulation in the presence of olanzapine 20 μM.

### 2.4. Inhibition of the Transcellular Transport of ASP^+^ by 5-HT_3_ Antagonists in OCT2/MATE1-Expressing MDCK Cells

**Figure 5:**
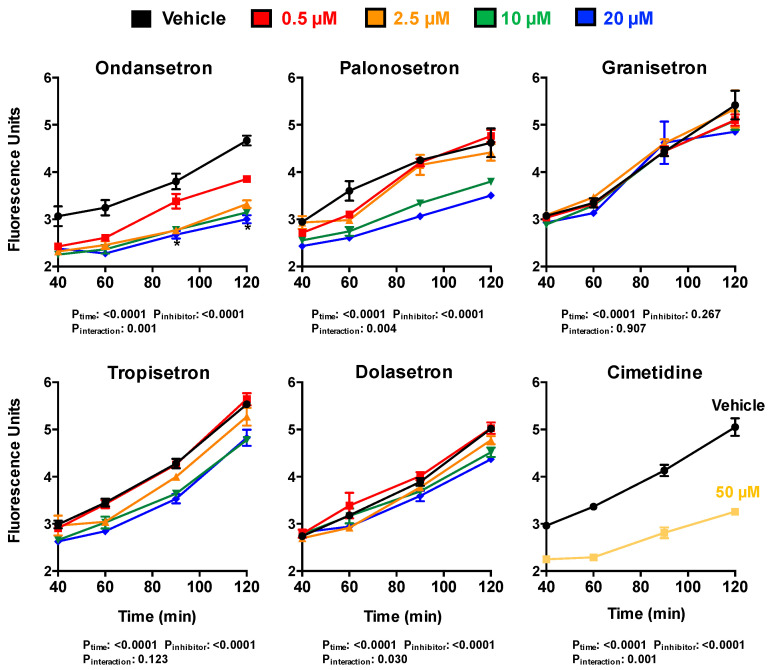
5-HT_3_ Antagonist Inhibition of ASP^+^ Transepithelial Transport in OCT2/MATE1-Transfected MDCK Cells. Cells were incubated with ASP^+^ (25 μM) in basolateral media and/or 5-HT_3_ antagonists (0.5–20 μM) or 50 μM cimetidine in apical and basolateral media for 120 min. Apical fluorescence was quantified between 40–120 min. Data are expressed as mean ± SE (*n* = 3). Two-way ANOVA analyses evaluated the influence of time and inhibitor on ASP^+^ transport.

**Figure 6:**
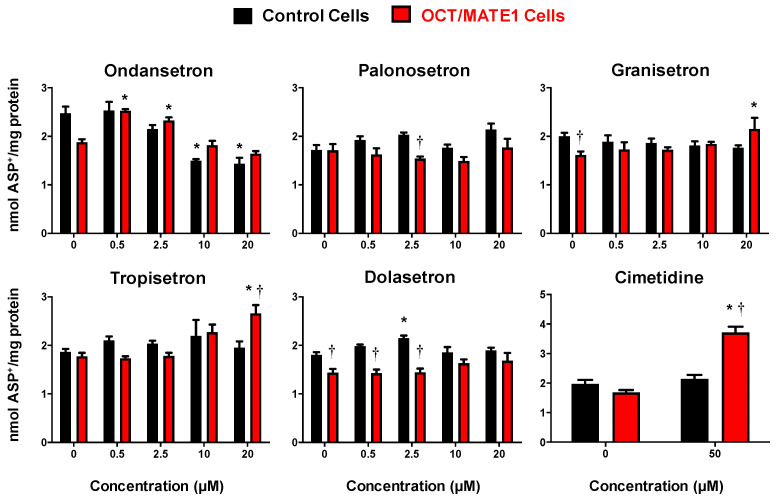
Intracellular Accumulation of ASP^+^ in OCT2/MATE1-Transfected MDCK Cells after 5-HT_3_ Antagonist Treatment. Empty vector (black bars) and OCT2/MATE1-transfected (red bars) MDCK cells were incubated with ASP^+^ (25 μM) in basolateral media with or without 5-HT_3_ antagonists (0.5–20 μM) or 50 μM cimetidine in apical and basolateral media for 120 min. Intracellular fluorescence (120 min) was quantified and normalized to protein concentration. Data are expressed as mean ± SE (*n* = 3). * *p* < 0.05 compared to no inhibitor. † *p* < 0.05 compared to empty vector control cells.

The five 5-HT_3_ antagonists (ondansetron, palonosetron, granisetron, tropisetron, and dolasetron) were evaluated for their ability to affect the transcellular transport and intracellular accumulation of ASP^+^ in OCT2/MATE1 double-transfected cells (Figure 5 and Figure 6). Cimetidine (50 μM) was included with each experiment in a parallel set of Transwells as a positive control. Interestingly, all five 5-HT_3_ antagonists exhibited varying degrees of inhibition on the transcellular B-to-A transport of ASP^+^ (Figure 5). Compared to vehicle-treated cells, ondansetron inhibited the B-to-A transport of ASP^+^ in a concentration-dependent manner, with 36% inhibition at 120 min in the highest concentration tested (20 μM). Palonosetron and tropisetron also displayed a concentration-dependent inhibition of ASP^+^ secretion, which was significant at 10 and 20 μM for all time-points. Inhibition of ASP^+^ secretion was observed with palonosetron and tropisetron at 20 μM. Dolasetron at 10 and 20 μM inhibited ASP^+^ transport at 120 min only. Lastly, granisetron did not alter the B-to-A transport of ASP^+^ at any concentration tested. Control cells showed no significant inhibition in the B-to-A transport of ASP^+^ with any of the 5-HT_3_ antagonists (data not shown).

### 2.5. ASP^+^ Intracellular Accumulation in OCT2/MATE1-Expressing MDCK Cells Following Treatment with 5-HT_3_ Antagonists

Low concentrations (0.5 and 2.5 μM) of ondansetron resulted in a 1.3-fold increase in intracellular ASP^+^ accumulation in OCT2/MATE1-expressing cells, while there was no difference compared to vehicle at higher concentrations (10 and 20 μM) (Figure 6). However, in control cells, there was a decrease (40%) in the accumulation of ASP^+^ at high concentrations of ondansetron. In OCT2/MATE1 cells, tropisetron and granisetron increased ASP^+^ accumulation (1.5 and 1.3-fold, respectively) at the highest concentration (20 μM). No significant changes in ASP^+^ accumulation were observed with palonosetron or dolasetron.

## 3. Discussion

OCT2 and MATE1 are key transporters that coordinate the renal secretion of organic cations. They share a number of overlapping substrates including metformin, cisplatin, lamivudine, and entecavir, as well as select 5-HT_3_ antagonist drugs [15,16,17,18,19,20,24]. Previous studies have suggested that 5-HT_3_ antagonists can also inhibit transport by OCT2 and MATE1. Most notably, ondansetron has been shown to reduce the transport of cisplatin and metformin by MATE1 [19]. The current study aimed to extend this prior work to compare five 5-HT_3_ antagonists for their ability to inhibit OCT2 and MATE1 individually when overexpressed in HEK293 cells and when coexpressed in MDCK cells and grown on Transwell inserts. Ondansetron and palonosetron were able to inhibit the uptake of a probe cationic chemical, ASP^+^, into HEK293 cells expressing OCT2 or MATE1, most notably, at concentrations as low as 0.1–0.5 µM. Similarly, granisetron, tropisetron, and dolasetron were also able to inhibit each transporter (at higher concentrations, however). Using OCT2/MATE1 double-transfected MDCK cells, ondansetron inhibited the basolateral-to-apical ASP^+^ secretion at all concentrations tested (0.5–20 μM), which was only observed at higher concentrations for palonosetron, tropisetron, and dolasetron. These data, obtained using a probe fluorescent substrate, suggest the potential for OCT2- and MATE1-mediated drug interactions with 5-HT_3_ antagonists.

The data generated in the OCT2/MATE1 double transfected cells largely agree with the HEK293 data, with the exception of granisetron. Ondansetron, palonosetron, and tropisetron exhibited concentration-dependent inhibition of the B-to-A transport of ASP^+^ with the order of potencies reflecting that observed with MATE1 inhibition seen in HEK293 cells. Surprisingly, granisetron did not exhibit any significant inhibition in OCT2/MATE1 MDCK cells, even at concentrations five times above the IC_50_ concentration observed in HEK293 cells expressing OCT2 or MATE1. Because the MDCK cells resemble native tubular cells to a greater extent than do HEK293 cells, there is the potential for the disposition of granisetron to be altered due to the expression of endogenous transporter orthologs of OCT2 or MATE1, or potentially other transporters. For example, MDCK cells highly express the canine P-glycoprotein transporter [25], which could lead to granisetron efflux. Supporting this speculation is the fact that a single nucleotide polymorphism in the human MDR1/ABCB1 transporter improved the clinical efficacy of granisetron for treating emesis [26]. These data suggest that granisetron may be a substrate for canine P-glycoprotein, causing an alteration in the intracellular concentration exposed to the MATE1 transporter.

While there is much overlap between the substrates and inhibitors of OCT and MATE transporters, distinctions do exist. Transporter-based drug–drug interactions can often be dependent upon the identity of the victim substrate being evaluated, as has been demonstrated for OCT2 [27,28,29]. For OCT2, selection of the cationic substrate has both a quantitative and qualitative impact on the extent and type of inhibition [27,28]. In contrast, the apparent Michaelis constant of the transported substrate and IC_50_ values for the inhibition of MATE1 across four different substrates was similar [30]. These data would suggest that there is a shared binding site for the interaction of substrates and inhibitors on the external surface of hMATE1. As a result, the interactions of 5-HT_3_ antagonist drugs with OCT2 and MATE1 may occur through different mechanisms, despite their shared cationic nature. Future studies are needed to advance the current study using a probe substrate and evaluating interactions with specific drug substrates such as cimetidine, metformin, and cisplatin.

Additional classes of drugs beyond the 5-HT_3_ antagonists are also used to prevent nausea and vomiting. In preliminary studies, we assessed the ability of other antiemetic drugs to inhibit OCT2 and MATE1-mediated transport of ASP^+^ in HEK293 cells. Interestingly, olanzapine, prochlorperazine, and metoclopramide inhibited OCT2 activity across a range of concentrations (Supplementary Figure S1). None of these drugs inhibited MATE1 activity at a concentration of 10 μM (data not shown). Other antiemetics, including aprepitant and dexamethasone, had no impact on ASP^+^ uptake in OCT2- or MATE1-expressing HEK293 cells (data not shown). As a result, there may be the potential for other antiemetic drugs (olanzapine, prochlorperazine, and metoclopramide) coadministered with 5-HT_3_ antagonists to also impact the renal secretion of cations, most notably through their ability to inhibit OCT2 uptake.

It is important to consider how well in vitro models recapitulate features of endogenous transport in human kidneys. Human OCT2 and MATE1 protein levels have been previously quantified in double-transfected MDCK cells by LC-MS/MS (hOCT2 and hMATE1 were 28.6 and 6.9 fmol/μg membrane protein, respectively) [31]. By comparison, studies determining the protein expression in the human kidney cortex and human kidney membranes reveal a more modest difference in expression between the two transporters or even greater MATE1 expression (OCT2 7.4 pmol/mg, MATE1 5.1 pmol/mg) [22] and (OCT2 5 fmol/μg, MATE1 10 fmol/μg) [32]. These differences between in vitro models and the endogenous human expression of OCT2 and MATE1 should be considered and factored into the development of physiologically-based pharmacokinetic models for potential cationic drug interactions in the kidneys.

The current study focused largely on the renal secretion of cationic drugs. However, it is important to recognize that the liver also expresses OCT1 and MATE1, which enable the biliary clearance of cationic chemicals. This is important because the 5-HT_3_ antagonists differ in their structure, metabolism, protein binding, and routes of clearance. Structurally, 5-HT_3_ receptor antagonists contain a basic amine, an aromatic or heteroaromatic ring system, and a carbonyl (or similar structure) that is coplanar to the aromatic region (Figure 1) [33]. Notably, the structure of the amine moiety differs for ondansetron and includes an imidazole, whereas 6-member rings are incorporated in the other 5-HT_3_ antagonists. The relevance of these structural differences on interaction with OCT2 and/or MATE1 require further investigation, but could explain the more significant effects of ondansetron at lower concentrations. In addition, some 5-HT_3_ antagonists are cleared extensively by the kidneys as parent or metabolite (such as palonosetron and tropisetron). Many of the 5-HT_3_ antagonists (including ondansetron, tropisetron, palonosetron, and granisetron) are highly metabolized (48–95%) by cytochrome P450 enzymes in the liver. Recently, studies have shown that metabolites may play a larger role in drug–drug interactions than previously thought [34]. Further testing of OCT2 and MATE1 transport with major metabolites of 5-HT_3_ antagonists that exceed 25% of parent systemic exposure is warranted. Likewise, within the chemical class, there are drugs with short half-lives (< 6 h, ondansetron and tropisetron), moderate half-lives (8–9 h, granisetron and dolasetron), and a long half-life (40 h, palonosetron). These factors should be considered when evaluating potential drug–drug interactions using in vitro transporter findings.

Based on the 2020 FDA Guidance for In Vitro Metabolism and Transporter-Mediated Drug–Drug Interaction Studies [35], a drug has the potential to inhibit the transporter in vivo if: the C_max_ (unbound)/IC_50_ value is ≥ 0.1. Based on these guidelines, the MATE1 C_max_/IC_50_ values for ondansetron indicate potential in vivo drug interactions using ASP^+^ as a probe substrate. Comparison of the IC_50_ values for the other 5-HT_3_ antagonists to their Cmax values (range from high nanomolar to low micromolar) would not suggest a risk of drug interaction, at least with ASP^+^ as the victim substrate. Likewise, we do not currently know the intrarenal concentrations of 5-HT_3_ antagonists, which would be important for evaluating potential MATE1 drug interactions. There are limited clinical data evaluating ondansetron-related drug interactions with OCT/MATE substrates. For example, levels of creatinine and metformin are increased in healthy subjects by ondansetron due to renal transporter inhibition [21,36]. Interestingly, ondansetron not only reduced renal clearance of metformin but also had improved glucose tolerance measures [21]. In other clinical studies, ondansetron has been reported to heighten the nephrotoxicity of a substrate drug by potentially altering transporter-mediated secretion within the kidney [37,38,39]. Together, these data warrant further investigation of ondansetron drug interactions through the inhibition of OCT and MATE transporters.

In summary, these in vitro data indicate that many of the 5-HT_3_ antagonists have a greater potency towards MATE1 inhibition, raising the potential for increased tubular concentration of cationic drugs. Further, ondansetron was potent enough to increase the intracellular concentration of a probe substrate, ASP^+^ at concentrations close to the clinically relevant C_max_. These data are in line with previous in vivo study in mice where increased cisplatin-mediated nephrotoxicity was observed with concurrent administration of ondansetron [19]. Based on the current criteria for evaluating clinical drug–drug interaction potential, the other 5-HT_3_ antagonists as well as antiemetic drugs tested in our study likely pose less risk of a clinically-relevant drug interaction due to much lower therapeutic plasma concentrations and higher inhibition constants.

## 4. Materials and Methods

### 4.1. Chemicals

4-(4-(Dimethylamino)styryl)-N-methylpyridinium iodide (ASP^+^) was purchased from Life Technologies (Grand Island, NY, USA). Tropisetron was purchased from Abcam (Cambridge, MA, USA). All other chemicals are from Sigma-Aldrich (St. Louis, MO, USA).

### 4.2. Cell Lines and Cell Culture

Empty Vector (EV) control (pcDNA5-transfected) and Flp-In human embryonic kidney (HEK)293 cell lines stably expressing human MATE1 and OCT2 transporters were generously provided by Dr. Kathy Giacomini at the University of California, San Francisco. HEK293 cells were cultured in Dulbecco’s Modified Eagle’s medium (DMEM) supplemented with 10% fetal bovine serum, 2 mM _L_-glutamine, 100 U/mL penicillin, 100 μg/mL streptomycin, and 200 μg/mL hygromycin B. Vector (pcDNA3.1+ and pcDNA3.1/Hygro(+)) and human OCT2/MATE1 double-transfected Madin–Darby canine kidney (MDCK) cell lines were generously provided by Dr. Joanne Wang at the University of Washington, Seattle, WA. MDCK cells were maintained in minimum essential medium (MEM) supplemented with 10% fetal bovine serum, 500 μg/mL G418 and 200 μg/mL hygromycin B. All cell lines were cultured in a humidified incubator at 37 °C with 5% CO_2_.

### 4.3. Uptake and Efflux Inhibition Assays in HEK293 Cells

OCT2- and MATE1-overexpressing HEK293 cells were seeded in clear poly-^D^-lysine-coated 24-well plates (Fisher Scientific, Hanover Park, IL, USA) and grown for 24 h until approximately 90% confluent. After washing once with prewarmed Hank’s Buffered Saline Solution (HBSS), cells were preincubated for 30 min at 37 °C with various 5-HT_3_ antagonists for OCT2 cells or in a 30 mM NH_4_Cl solution in HBSS at pH 6.5 for MATE1 cells for intracellular acidification. Uptake into OCT2 cells was initiated through exposure to 10 μM of fluorescent substrate ASP^+^ directly in the incubation media. Uptake into MATE1 cells was initiated by application of HBSS at pH 7.4 containing 5-HT_3_ antagonists and 10 μM of fluorescent substrate ASP^+^. After incubating for 1 min at 37 °C on a shaker, substrate uptake was stopped by adding ice-cold HBSS containing 500 μM cimetidine. Media was removed and washed four times with ice-cold HBSS. Cells were lysed with 1% Triton X-100. Fluorescence was detected using a Spectramax Microplate Reader (Molecular Devices, Sunnyvale, CA, USA) at the following wavelengths (Excitation 485 nm/Emission 495 nm). Intracellular fluorescence was normalized to total protein concentration of cell lysates from each well using the bicinchoninic acid (BCA) assay (Pierce Biotechnology, Rockford, IL, USA). Experiments were repeated three separate times, with three to four replicates in each experiment.

### 4.4. Transwell Studies in MDCK-OCT2/MATE1 Cells

Control and OCT2/MATE1-expressing MDCK cells were evaluated for protein expression of OCT2 and MATE1 using SDS-PAGE and western blotting with specific primary antibodies (OCT2, sc292622 1:500 and MATE1, sc133390 1:250, Santa Cruz Biotechnology, Santa Cruz, CA, USA), followed by an antirabbit HRP-conjugated secondary antibody (1:1000, Sigma Aldrich, St. Louis, MO, USA) and Super Signal Western Dura Extended Duration Substrate (Pierce Biotechnology, Rockford, IL, USA). Detection was performed with a FluorChem imager (ProteinSimple, Santa Clara, CA, USA). Both MDCK cell lines were seeded on 0.4 μm transwell inserts (VWR, Radnor, PA, USA) at a density of 2 × 10^5^ cells/cm^2^. Transport experiments were performed 3 to 5 days after seeding. The integrity of MDCK monolayers was verified by measuring transepithelial electrical resistance (TEER) >150 Ω*cm^2^ using an epithelial voltohmmeter, EVOM^2^ (World Precision Instruments, Sarasota, FL). Proper formation of tight junctions was also verified by measurement of passive permeability of lucifer yellow in the basolateral-to-apical (B-to-A) direction. Lucifer yellow (20 μM) was applied to the basolateral chamber for 1 h, and media were collected from the apical chamber. Lucifer yellow fluorescence was read at an excitation wavelength of 430 nm and emission wavelength of 538 nm. Average passive permeability (P_app_) values were 7 × 10^−7^ cm/s, which is line with literature values [40].

After washing the cells once with Hank’s Buffered Saline Solution (HBSS) pH 7.4, transport studies were initiated after aspirating the wash buffer from both the apical and basal chambers. Cells were incubated with 5-HT_3_ antagonists in the apical chamber in HBSS pH 6.0 and 5-HT_3_ antagonists with ASP^+^ (25 μM) in the basolateral chamber in HBSS pH 7.4 and incubated at 37 °C for 120 min. To measure time-dependent transcellular transport, an aliquot of the incubation medium (100 μL) from the apical chamber (receiving chamber) was collected at 40, 60, 90, and 120 min and replaced with an equal volume of fresh buffer containing 5-HT_3_ antagonist or the positive control chemical at the original concentration. After 120 min, treatment media was removed and Transwells were washed three times with ice-cold HBSS. Cells were lysed with 1% Triton X-100. Fluorescence was detected using Spectramax Microplate Reader at the following wavelengths (Excitation 485 nm/Emission 495 nm). Intracellular fluorescence was normalized to total protein concentration of cell lysates from each transwell using the BCA assay. Experiments were performed in three individual Transwell inserts.

### 4.5. Statistical Analysis

GraphPad Prism v6 (GraphPad Software, La Jolla, CA, USA) was used for statistical analysis. K_m_ and V_max_ were calculated using nonlinear regression (Michaelis–Menten enzyme kinetics equation, (Y = Vmax × X/(Km + X), fit for least squares). Data with two variables were analyzed using a two-way ANOVA followed by a one-way ANOVA and/or Dunnett’s post-hoc test for multiple comparisons. IC_50_ values were calculated via a nonlinear regression to fit least squares. Differences were considered statistically significant at *p* < 0.05.

## Figures and Tables

**Table 1:** 5-HT_3_ antagonist inhibition of in vitro ASP^+^ transport by OCT2 and MATE1 in HEK293 cells ^1^.

5-HT_3_ Antagonist	OCT2 IC_50_ (µM)	MATE1 IC_50_ (µM)
Ondansetron	2.6 ± 0.9	0.1 ± 0.1
Palonosetron	2.2 ± 0.3	1.6 ± 0.6
Granisetron	3.8 ± 1.6	5.0 ± 1.1
Tropisetron	31.3 ± 6.6	1.6 ± 0.9
Dolasetron	85.4 ± 3.4	27.4 ± 2.8
Cimetidine	24.5 ± 4.0	0.23 ± 0.2

^1^ Avg ± SE (*n* = 3).

## Data Availability

Not applicable.

## Supplementary Materials

The following are available online at https://www.mdpi.com/article/10.3390/ijms22126439/s1.
