# Supplementary material for: In Vitro Inhibition of Renal OCT2 and MATE1 Secretion by Antiemetic Drugs
Source: Int J Mol Sci. 2021 Jun 16;22(12):6439. doi: 10.3390/ijms22126439 (PMC8234231; doi:10.3390/ijms22126439)
Supplement: Supplementary file 1 [file ijms-22-06439-s001.zip › ijms-1221474-supplementary.pdf]

SUPPLEMENTARY INFORMATION

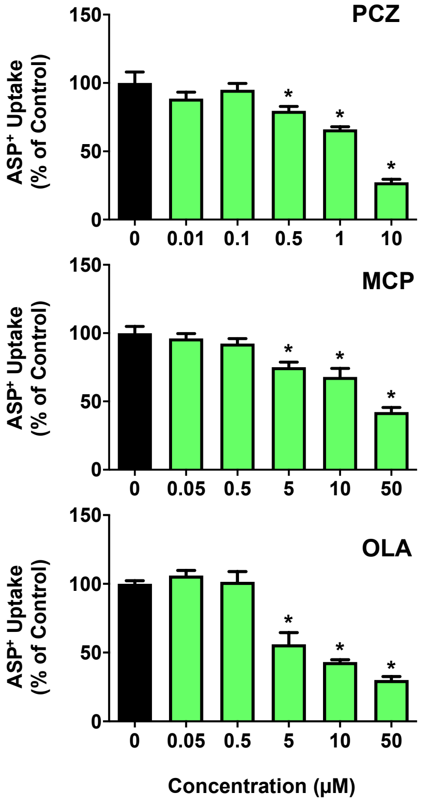

Figure S1. Inhibition of ASP^+ Transport by Other Antiemetic Drugs. HEK293 cells overexpressing OCT2 were incubated with ASP^+ (10 μM) in the presence and absence of other antiementic drugs (OLA, olanzapine; MCP, Metoclopramide; PCZ, Prochlorperazine). Fluorescence was quantified and normalized to protein concentration. Data are expressed as mean ± SE (n = 3). * $p < 0.05$ compared to vehicle control (0 μM).
